# Comparative Effectiveness of Pharmacological and Non-pharmacological Interventions for Hypertension Management: A Systematic Review and Meta-Analysis

**DOI:** 10.7759/cureus.94130

**Published:** 2025-10-08

**Authors:** Bandar A Almabruk, Lana S Alturki, Abdullah M Alghamdi, Turki S Almutairi, Eman A Alsulami, Ahad S Alsharif, Lamiaa Saad, Showq A Alsaedi, Ola E Alkhoshiban, Nouf Alshareef, Loai A AlRabaie, Abeer Alsharif, Layan Y Khan, Talal W Bakhsh

**Affiliations:** 1 Department of Internal Medicine, King Salman Hospital Riyadh, Riyadh, SAU; 2 School of Medicine, Sulaiman Alrajhi University, Riyadh, SAU; 3 Department of Internal Medicine, Faculty of Medicine, Albaha University, Albaha, SAU; 4 College of Medicine and Surgery, Taif University, Taif, SAU; 5 Department of Medicine, King Abdulaziz University Faculty of Medicine, Jeddah, SAU; 6 Faculty of Medicine, Taibah University, Madina, SAU; 7 College of Medicine, Vision College, Riyadh, SAU; 8 Department of Internal Medicine, Prince Mohammed Bin Abdulaziz Hospital, National Guard Health Affairs, Madinah, SAU; 9 Unaizah College of Medicine and Medical Science, Qassim University Qassim, Qassim, SAU; 10 Department of Internal Medicine, King Abdulaziz University Hospital, Jeddah, SAU; 11 Department of Internal Medicine, Ibn Sina National College, Jeddah, SAU; 12 Department of Pharmacology, King Abdulaziz Hospital, National Guard, Jeddah, Jeddah, SAU; 13 Department of Internal Medicine, College of Medicine, King Abdulaziz University, Jeddah, SAU; 14 Department of Medicine, King Saud Bin Abdulaziz University for Health Sciences, Jeddah, SAU

**Keywords:** antihypertensive drugs, blood pressure, hypertension, lifestyle modification, non-pharmacological interventions, pharmacological therapy

## Abstract

Hypertension is a major global health burden, which is strongly linked to stroke, heart disease, kidney failure, and premature death. Pharmacological therapies remain the cornerstone of management, but adherence, cost, and side effects pose significant challenges. Lifestyle interventions such as diet, exercise, and stress reduction provide comparable benefits with fewer risks. We conducted a PRISMA-guided systematic review (2015-2025) across PubMed and Google Scholar. The eligible studies included adult hypertensive patients receiving pharmacological or non-pharmacological interventions. Our review of 21 studies (n > 63,000) showed consistent blood pressure (BP) benefits across intervention types. The pooled systolic BP reduction was -6.95 mmHg (95% CI: 4.79-10.09) across all the studies, with the pharmacological interventions (-6.83 mmHg), non-pharmacological (-6.03 mmHg), and combined (-8.37 mmHg) interventions yielding comparable effects. The diastolic reductions were greatest with the non-pharmacological interventions (-6.77 mmHg), compared with pharmacological (-2.52 mmHg) and combined (-3.34 mmHg). The target BP achievement was highest with pharmacological therapy (67.8%), followed by the combined (58.9%) and the non-pharmacological (26.4%) approach only. The mortality was lower with pharmacological management (3.7%) compared to combined interventions (14%, single study). The secondary outcomes included modest BMI reduction (-0.80 kg/m²) and cholesterol lowering (-7.04 mg/dL), with the greatest effects from combined strategies. This study shows that both pharmacological and non-pharmacological interventions effectively reduce BP, with the combined strategies showing the greatest impact. Pharmacological therapies achieved superior target control and lower mortality, while lifestyle changes improved metabolic outcomes.

## Introduction and background

Hypertension is one of the most common chronic conditions and the greatest health burden worldwide. It is the major cause of preventable illness and death [1]. According to the WHO, an estimated 1.28 billion adults aged 30-79 years worldwide have hypertension, with most (two-thirds) living in low- and middle-income countries [2]. High blood pressure (BP) is strongly linked to conditions such as stroke, heart disease, kidney failure, and premature mortality [3]. Despite progress in the diagnosis and treatment, global control rates of this condition remain low. Many patients do not achieve recommended targets, and this highlights the importance of evaluating the range of available strategies for effective BP management.

Pharmacological management or drug therapy has been the foundation for hypertension control for decades [4]. Several classes of medicines, which include diuretics, angiotensin-converting enzyme inhibitors (ACEI), angiotensin receptor blockers (ARBS), calcium channel blockers (CCBs), and beta blockers, have proven beneficial [5]. Previous large trials confirm that these drugs reduce BP and lower the risk of the complications of diabetes, such as cardiovascular events or stroke [6]. However, their use in the routine practice is affected by cost, side effects, drug interactions, and the challenge of long-term adherence. In older patients, polypharmacy is common and can complicate the management of hypertension [7]. These limitations mean that drug therapy alone is often not enough to the achieve good control at the population level.

Furthermore, the non-pharmacological interventions play an equally important role in the management of hypertension. Lifestyle measures, which included dietary change, reduced salt intake, regular physical activity, weight control, moderation of alcohol, and stress reduction, all contribute to lowering BP [8]. Moreover, structured programs, such as the DASH (Dietary Approaches to Stop Hypertension) diet or the aerobic exercise routines, can reduce the systolic and the diastolic pressure by amounts similar to the single-drug therapy in some studies [9]. These approaches also improve metabolic health, which reduces the risk of obesity and diabetes, and support overall well-being. Because they have minimal adverse effects, they are suitable for all age groups and may also increase patient involvement in care.

With several options available, clinicians often need to choose the best combination of treatments for individual patients. Guidelines recommended that lifestyle measures should be advised for all patients and that drugs should be added when non-pharmacological interventions are unable to achieve desirable results [9]. However, there are some questions that still exist about the efficacy of non-pharmacological and pharmacological interventions and whether combinations of both give additional benefit. Evidence from younger patients, from those with comorbidities, and from low-resource settings is still limited. This systematic review and meta-analysis allow comparison across trials and observational studies. By pooling data, it is possible to quantify the effect of different pharmacological and non-pharmacological strategies and examine differences between patient groups. These methods can also provide information on adherence, safety, and cost effectiveness, which are essential for real-world application.

The aim of this study is to systematically review and synthesize evidence about the comparative effectiveness of pharmacological and non-pharmacological interventions for the management of hypertension. This study seeks to provide a comprehensive evaluation of the available strategies, which will highlight their relative strengths and limitations. It will provide evidence-based recommendations for improving BP control. By clarifying the role of both pharmacological and lifestyle approaches, this review will support efforts to reduce the global burden of hypertension and its complications.

## Review

Methodology

Study Design

This review followed the principles set out in the Preferred Reporting Items for Systematic Reviews and Meta-Analyses (PRISMA) 2020 guidelines.

Search Strategy

To capture as much relevant evidence as possible, we carried out a broad search across several electronic databases, namely PubMed and Google Scholar. The searches covered articles from 2015 to 2025. We used a combination of keywords and subject headings relating to hypertension, pharmacological treatment (including specific drug classes), and non-pharmacological strategies such as diet, exercise, and lifestyle changes. The search strings were adapted slightly for each database. In addition, we checked the references of relevant reviews and included articles to identify any studies that may have been missed during the electronic search.

Eligibility Criteria

Studies were included if they enrolled adults aged 18 years or older with a diagnosis of hypertension and compared either pharmacological interventions (antihypertensive drugs of any class) or non-pharmacological approaches (for example, dietary modification, structured exercise programs, weight control, or stress reduction) with a comparator group. Eligible designs were randomized controlled trials (RCTs), cluster randomized trials, or other controlled clinical studies. We excluded studies focusing on children, pregnant women, or animal models, as well as those without full text or published in languages other than English.

Study Selection

**Figure 1:**
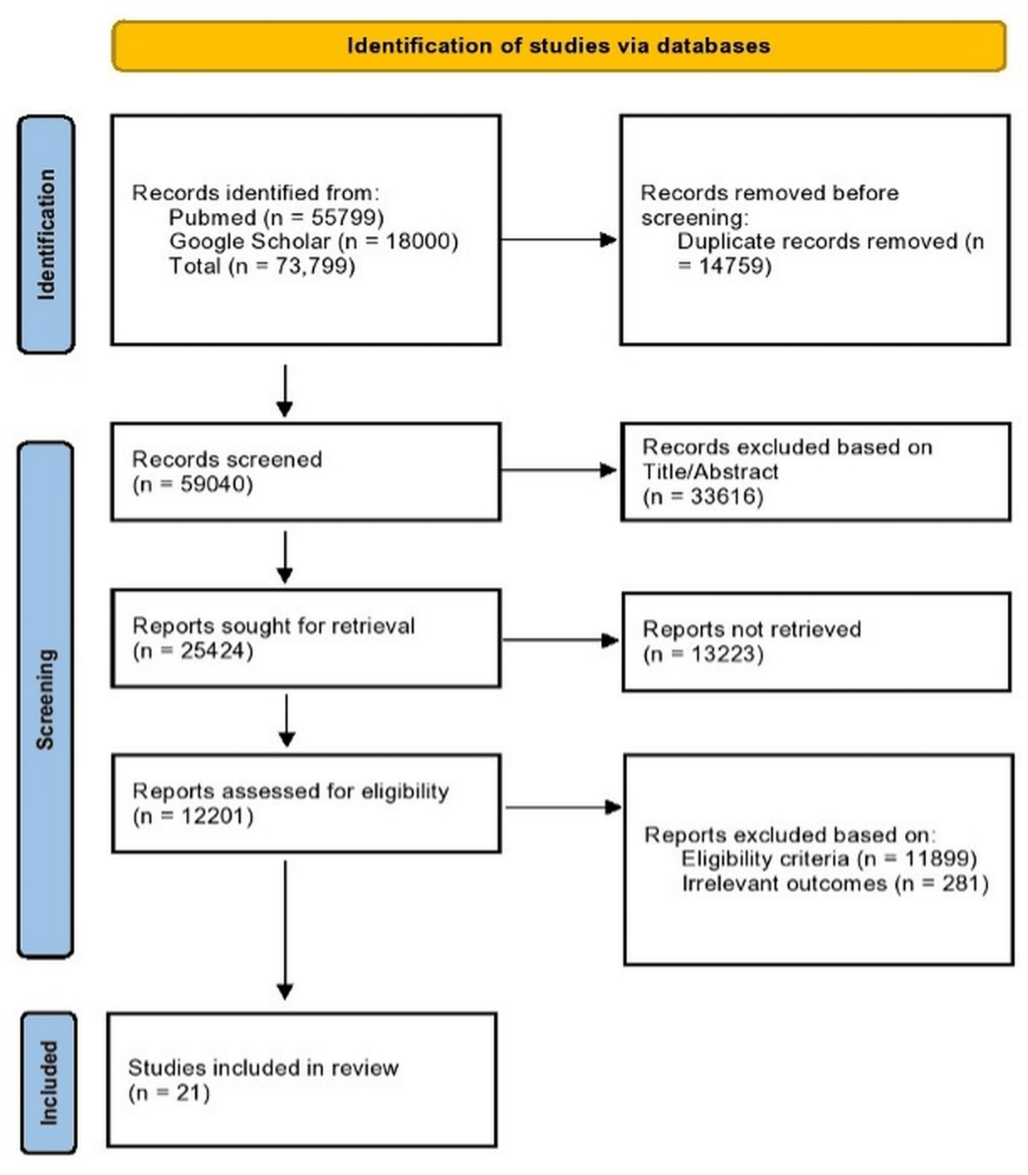
PRISMA chart showing selection and inclusion of studies [10]

All retrieved references were exported into EndNote (Clarivate Analytics), where duplicates were removed. The remaining records were screened independently by two reviewers in two stages. First, titles and abstracts were checked for relevance. Second, full texts of potentially eligible papers were assessed against the inclusion and exclusion criteria. Disagreements were resolved by discussion and, if necessary, consultation with a third reviewer. The process of study selection was documented using a PRISMA flow diagram (Figure 1).

Assessment of Agreement

The inter-reviewer agreement was evaluated using the kappa (κ) statistic for screening. A priori classification was defined according to the following criteria: κ of 0.91-0.99 was considered almost perfect agreement, κ of 0.71-0.90 was considerable agreement, k of 0.61-0.70 was high agreement, κ of 0.41-0.60 was moderate agreement, κ of 0.21-0.40 was fair agreement, and κ or ICC value of 0.20 or less was classified as no agreement.

Data Extraction

The data from the final included studies were independently extracted by the two reviewers using a standardized Excel form. The extracted variables were the study design, year, sample size, and geographic setting, along with the participant characteristics such as age, sex, baseline BP, and comorbidities. The intervention details were classified as both pharmacological (ACEIs, ARBs, beta blockers, CCBs, diuretics) and non-pharmacological (diet, exercise, weight control, lifestyle modification), or a combination of both. The outcomes included changes in the systolic/diastolic blood pressure (SBP/DBP), the target BP achievement, mortality, cardiovascular events, hospitalizations, adverse events, BMI, lipids, HbA1c, quality of life, and the adherence rate of both management. The discrepancies were resolved by consensus, and all the data were verified before synthesis.

Results

Study Selection

In total, the search identified 73,799 records. After removing duplicates, 59,040 studies remained for screening. Of these, 33,616 were excluded on the basis of title and abstract. Full texts were obtained for 12,201 articles, and following a detailed review, 21 studies satisfied all inclusion criteria and were incorporated into the final analysis (Figure 1).

Risk of Bias and Quality Assessment

**Figure 2:**
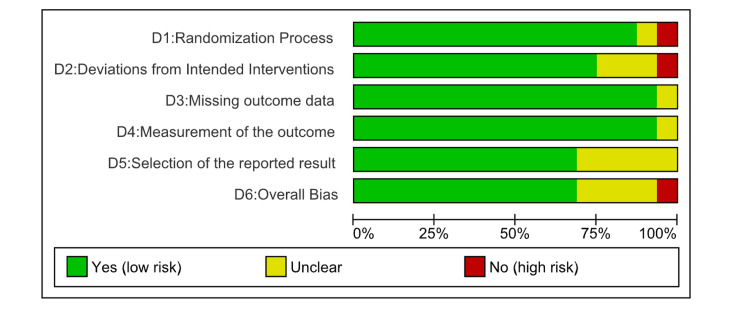
Risk-of-bias graph: review authors' judgements about each risk of bias item presented as percentages across all included studies (RoB2). ROB2, Cochrane Risk of Bias 2.0

**Figure 3:**
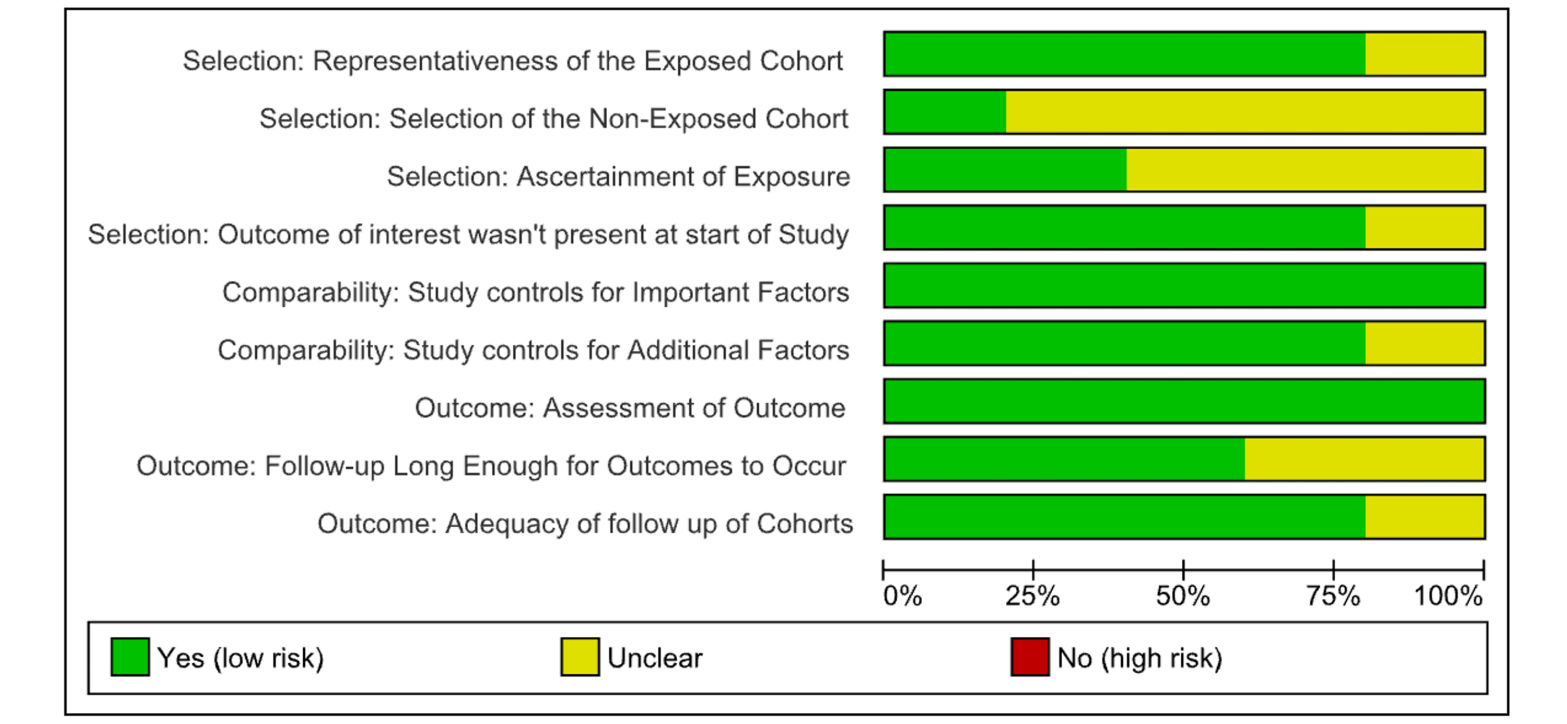
Risk-of-bias graph: review authors' judgements about each risk of bias item presented as percentages across all included studies (NOS). NOS, Newcastle-Ottawa Scale

The methodological quality of the included studies was thoroughly assessed with the help of two validated tools: the Newcastle-Ottawa Scale (NOS) for observational studies and the Cochrane Risk of Bias 2.0 (ROB 2) tool for the RCTs. This evaluation was conducted independently by three reviewers. Any disagreements were resolved through consensus, and, if needed, a senior reviewer was consulted to ensure objectivity. For the RCT studies, most studies showed a low risk of bias in certain domains such as measurement of outcomes and missing outcome data (Figures 2, 3).

**Figure 4:**
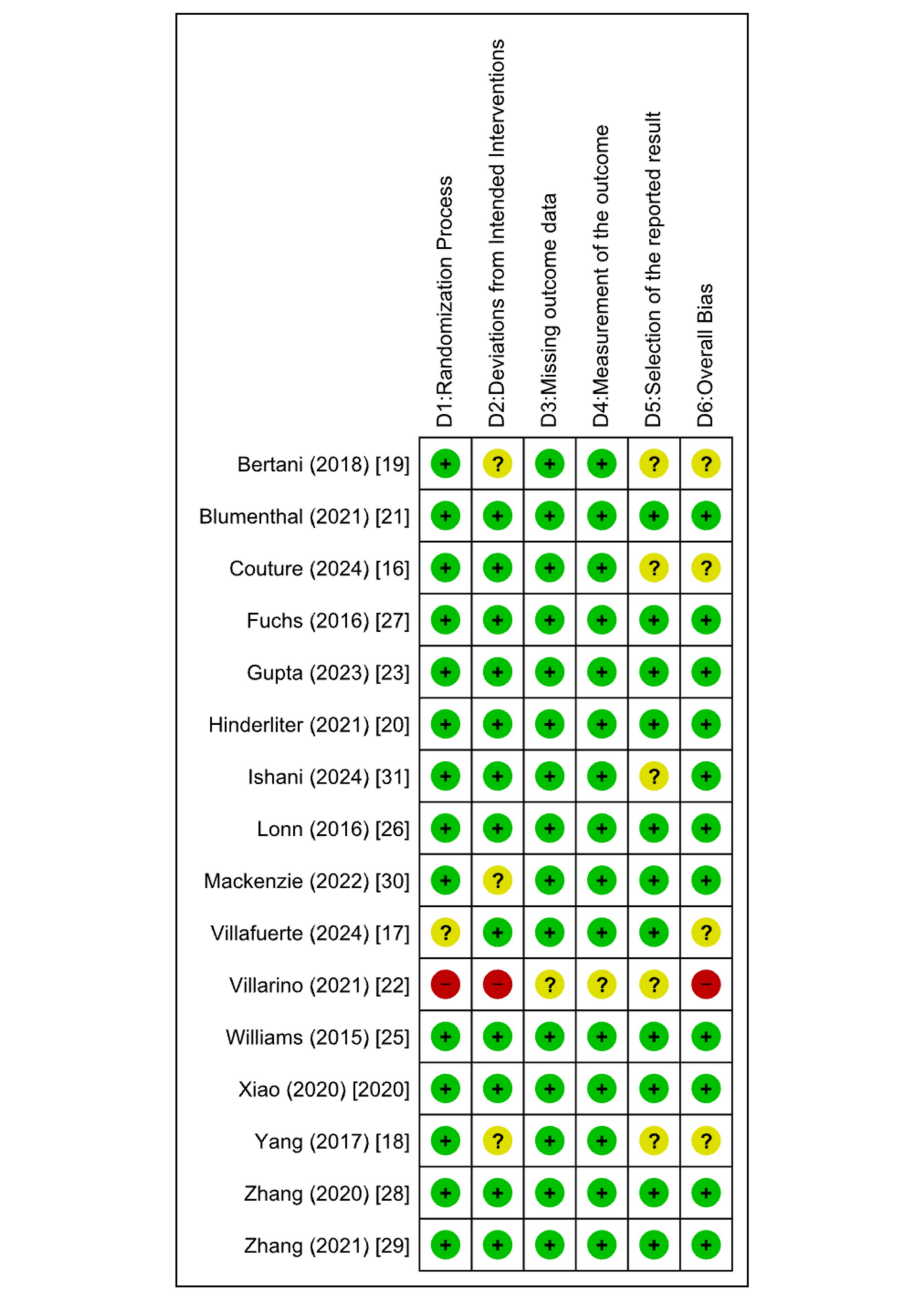
Risk-of-bias summary: review authors' judgements about each risk of bias item for each included study (RoB2). ROB2, Cochrane Risk of Bias 2.0

**Figure 5:**
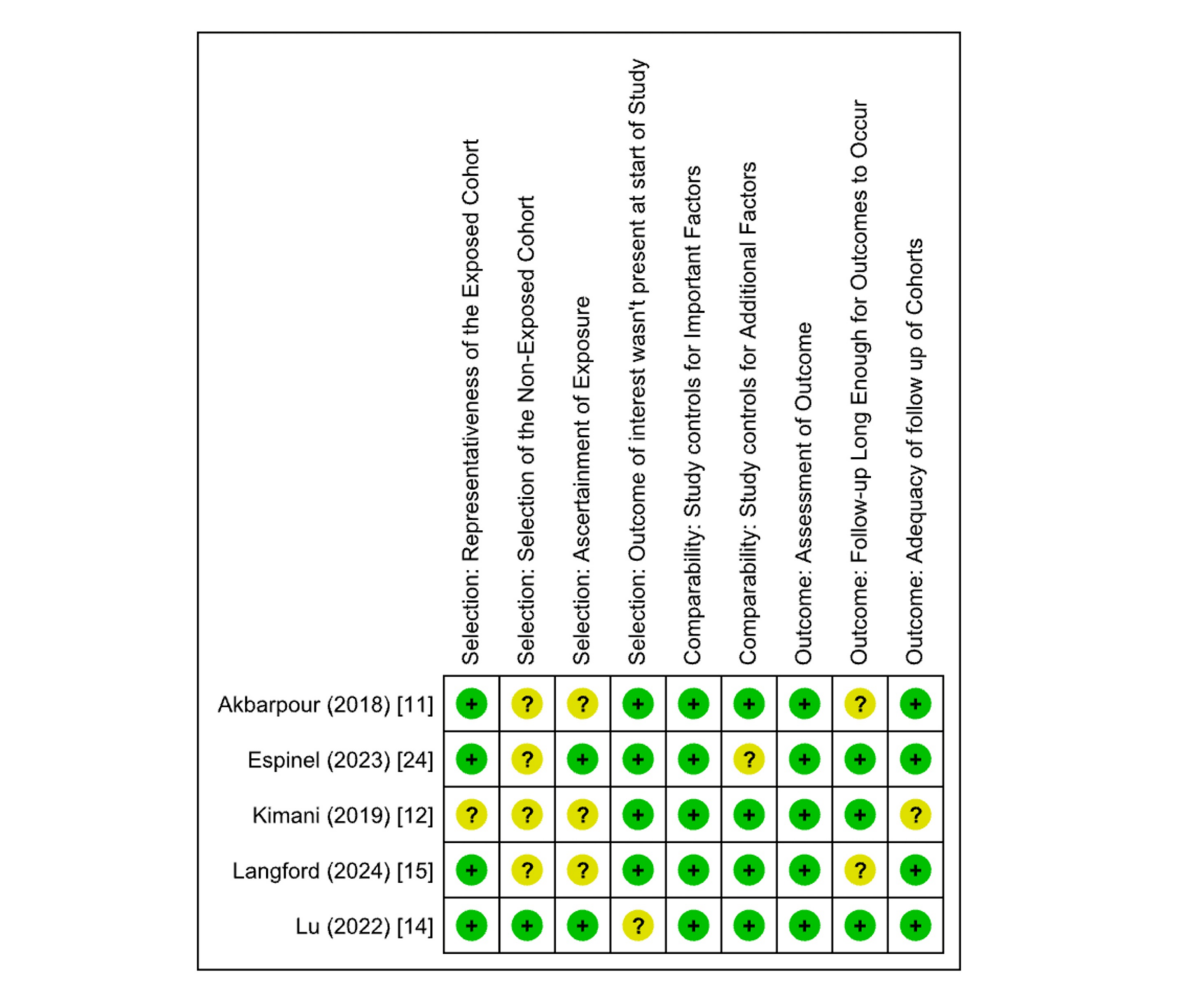
Risk-of-bias summary: review authors' judgements about each risk of bias item for each included study (NOS). NOS, Newcastle-Ottawa Scale

Observational studies assessed using the NOS showed low-to-moderate risk of bias in different domains (Figures 4, 5).

Characteristics of the Included Studies

Our review included 21 studies [11-31], which reflected the wide geographic and methodological diversity, which ranged from community surveys to large RCTs. Cross-sectional studies provided baseline insights, such as the study by Akbarpour et al. [11] in Iran with 2,577 participants, Kimani et al. [12] in Kenya with 229 participants, Langford et al. [15] in New York with 209 participants, and Espinel et al. [24] in Spain with 30 participants. Most included studies were RCTs of varying scale. In Asia, Xiao et al. [13] enrolled 2,912 participants in rural Jiangsu, China, while Zhang et al. [28] and Zhang et al. [29] conducted large multicenter Chinese trials with 14,978 and 8,511 participants, respectively. Lu et al. [14] added long-term cohort data from 14,392 factory retirees.

In Western countries, Williams et al. [25] ran an RCT across 14 UK sites, and Mackenzie et al. [30] reported one of the largest trials, enrolling 21,104 participants through a National Health Service-linked platform. Similarly, Ishani et al. [31] studied 13,523 Veterans Affairs (VA) patients in the USA. There are several medium-sized trials that targeted the shorter interventions, including those by Yang et al. [18] in Korea, Bertani et al. [19] in Brazil, and Hinderliter et al. [20] and Blumenthal et al. [21] in the USA. The multinational evidence was added by Lonn et al. [26] with 12,705 participants across 21 countries, while Villarino et al. [22] provided quasi-experimental data from the Philippines (Table 1).

**Table 1:** Characteristics of the included studies KNH, Kenyatta National Hospital; NHS, National Health Service; NYC, New York City; Quasi-exp., quasi-experimental; RCT, randomized controlled trial; USP, Universidade de São Paulo; VA, Veterans Affairs

Author (Year)	Country/Region	Design	Setting	Follow-Up	Sample (N)
Akbarpour et al. (2018) [11]	Iran	Cross-sectional	Community survey	N/A	2,577
Kimani et al. (2019) [12]	Kenya	Cross-sectional	Referral hospital (KNH, Nairobi)	N/A	229
Xiao et al. (2020) [13]	China (Jiangsu)	RCT	Rural community	1 yr	2,912
Lu et al. (2022) [14]	China	Cohort	Factory retirees	Median 7.3 yrs	14,392
Langford et al. (2024) [15]	USA (NYC + registry)	Cross-sectional	Health system + research match	N/A	209
Marin-Couture et al. (2024) [16]	Canada	RCT	Primary care (Université Laval)	6 Months	51
Unda Villafuerte et al. (2024) [17]	Spain	RCT	Primary care centers	6 Months	141
Yang et al. (2017) [18]	Korea	RCT	Hospital family practices	12 Weeks	1,139
Bertani et al. (2018) [19]	Brazil	RCT	Teaching health center (USP)	12 Weeks	61
Hinderliter et al. (2021) [20]	USA	RCT	Community/research center	16 Weeks	144
Blumenthal et al. (2021) [21]	USA	RCT	Cardiac rehab/community	4 Months	140
Villarino et al. (2021) [22]	Philippines	Quasi-exp.	Rural health unit	10 Months	50
Gupta et al. (2023) [23]	USA	RCT	Community participants	2 Weeks	213
Espinel et al. (2023) [24]	Spain	Cross-sectional	Hospital outpatient	6 Months	30
Williams et al. (2015) [25]	UK	RCT	12 secondary + 2 primary sites	1 yr	335
Lonn et al. (2016) [26]	21 countries	RCT	Multicenter (228)	Median 5.6 yrs	12,705
Fuchs et al. (2016) [27]	Brazil	RCT	21 academic centers	18 Months	730
Zhang et al. (2020) [28]	China	RCT	32 communities	Median 4.5 yrs	14,978
Zhang et al. (2021) [29]	China	RCT	42 clinical centers	Median 3.34 yrs	8,511
Mackenzie et al. (2022) [30]	UK	RCT	Decentralized, NHS-linked	Median 5.2 yrs (max 9.3)	21,104
Ishani et al. (2024) [31]	USA	RCT	VA healthcare (outpatient)	Mean 2.4 ± 1.4 yrs	13,523

Characteristics of Participants

Table 2 shows the baseline characteristics of participants across the included studies. In Akbarpour et al.’s study [11], 52.9% were aware and 47.1% were not aware of the intervention, while in Xiao et al.’s study [13], 66.8% were in the intervention group versus 33.2% in the control group. Larger cohorts were reported by Lu et al. [14] with 10,624 interventions and 3,768 controls, and Mackenzie et al. [30] with over 21,000 participants. Smaller trials were reported by Marin-Couture et al. [16] with group allocations of 12-13 and Espinel et al. [24] with 30 participants.

**Table 2:** Characteristics of participants in the included studies Biso, bisoprolol; CABG, coronary artery bypass graft; Cand+HCTZ, candesartan + hydrochlorothiazide; CA, continuous aerobic; CHD, coronary heart disease; CKD, chronic kidney disease; COMBI, combined intervention; COPD, chronic obstructive pulmonary disease; Ctrl, control; CTD, chlorthalidone; CVD, cardiovascular disease; DASH; Dietary Approaches to Stop Hypertension; DBP, diastolic blood pressure; DM, diabetes mellitus; Dox, doxazosin; Dysgly, dysglycemia; Dyslip, dyslipidemia; FA, folic acid; FamHx, family history; HCTZ, hydrochlorothiazide; HF, heart failure; HL, hyperlipidemia; HTN, hypertension; IA, interval aerobic; Int, intervention; LVH, left ventricular hypertrophy; MED, standard care/control; MetS, metabolic syndrome; MI, myocardial infarction; MP, Mediterranean pattern; N/A, not available; NMP, non-Mediterranean pattern; Normo, normotensive; NUT, nutrition/DASH; OSA, obstructive sleep apnea; PA, physical activity; PCI, percutaneous coronary intervention; Plaque, carotid artery plaque; R, resistance training; SBP, systolic blood pressure; Spiro, spironolactone; WM, weight management

Author (Year)	Intervention (N, %)	Control (N, %)	Mean Age (SD/range)	Sex (M/F (N, %)	Baseline SBP (Mean ± SD)	Baseline DBP (Mean ± SD)	HTN Type	Baseline BMI (mean ± SD)	Comorbidities (N, %)
Akbarpour et al. (2018) [11]	Aware: 1,364 (52.9%)	Not aware: 1,213 (47.1%)	40.8 yrs (95% CI 40.6–41.0)	M: 1,293 (50.2%), F: 1,284 (49.8%)	136.4 vs 139.1 (Total 138.0)	85.0 vs 92.5 (Total 89.6)	N/A	“Good” (18.5–25): 27.3%	N/A
Kimani et al. (2019) [12]	N/A	N/A	40.2% <50 yrs; 67.3% <60 yrs	M: 102 (44.5%), F: 127 (55.5%)	139.5 ±14.1	89.2 ±12.8	Essential	28.9 ±4.2	Obesity more in females
Xiao et al. (2020) [13]	1,945 (66.8%)	967 (33.2%)	Int: 57.7 ±7.5; Ctrl: 58.1 ±8.9	Int: M 829 (42.6%), F 1,116 (57.4%); Ctrl: M 380 (39.3%), F 587 (60.7%)	150.4 ±11.4 vs 151.1 ±17.4	94.5 ±7.2 vs 94.6 ±8.5	Essential	N/A	FamHx: 16.9 vs 17.8%; CHD: 1.5 vs 2.5%; Stroke: 1.4 vs 2.4%; DM: 1.4 vs 2.1%
Lu et al. (2022) [14]	10,624 (73.8%)	3,768 (26.2%)	65.6 ±7.4	M: 7,277 (50.6%), F: 7,115 (49.4%)	148–142 (by group)	86–81 (by group)	N/A	N/A	DM: 20.9–31.9%; CVD: 19–39.9%
Langford et al. (2024) [15]	Lifestyle changes only	N/A	44.7 ±14.7	M: 92 (44%), F: 117 (56%)	N/A	N/A	N/A	N/A	N/A
Marin-Couture et al. (2024) [16]	PA (13), NUT (12), COMBI (13)	MED (13)	NMP: 50.3 ±2.3; MP: 58.8 ±2.6	N/A	MED: 137.2; PA: 134.1; NUT: 143.2; COMBI: 130.3	MED: 81.6; PA: 78.3; NUT: 83.3; COMBI: 80.2	Stage 1	MED: 28.8; PA: 32.7; NUT: 29.3; COMBI: 35.0	Excluded DM, CKD <30, CVD
Unda Villafuerte et al. (2024) [17]	70 (49.6%)	71 (50.4%)	Int: 59.9 ±9.0; Ctrl: 60.7 ±8.9	Int: M 32 (45.7%), F 38 (54.3%); Ctrl: M 35 (49.3%), F 36 (50.7%)	146.7 ±13.9 vs 142.9 ±14.6	89.4 ±8.3 vs 86.3 ±9.0	Essential, severe in 2 (2.9%)	Int: 30.0 ±6.5; Ctrl: 30.6 ±4.9	DM: 14–17%; Dyslip: ~61%; Obesity: ~53%; Smokers: 25–33%
Yang et al. (2017) [18]	N/A	N/A	64.9 ±10.2	M: 449 (39.4%), F: 690 (60.6%)	N/A	N/A	N/A	25.1 ±3.0	Comorbid present: 644 (56.5%)
Bertani et al. (2018) [19]	Aerobic CA (15), IA (15), R (16)	Control (15)	CA: 67.3 ±5.4; IA: 68.1 ±5.8; R: 67.7 ±5.8; Ctrl: 66.6 ±5.2	M: 20 (32.8%), F: 41 (67.2%)	CA: 129.5; IA: 131.6; R: 126.9; Ctrl: 130.9	CA: 75.5; IA: 78.6; R: 73.6; Ctrl: 77.2	Elderly HTN	N/A	Excluded DM, CKD, severe CVD
Hinderliter et al. (2021) [20]	DASH+WM: 49 (34%); DASH: 46 (32%)	Usual care: 49 (34%)	52 ±10 (≥35 yrs)	M: 47 (33%), F: 97 (67%)	138 ±9	86 ±6	Stage 1	33.1 ±3.9	DM: 1 (<1%); Smoking: 10 (7%)
Blumenthal et al. (2021) [21]	90 (64%)	50 (36%)	63 ±9	M: 73 (52%), F: 67 (48%)	139 ±10	79 ±9	Resistant	36 ±6	DM: 31%; CKD: 24%; CVD: 11%; Obesity: most
Villarino et al. (2021) [22]	50	N/A	30–89 yrs (grouped)	M: 11 (22%), F: 39 (78%)	146.5 ±19.6	84.6 ±12.6	Stage 1–2	N/A	N/A
Gupta et al. (2023) [23]	Low-Na: 95 (45%)	High-Na: 118 (55%)	Median 61 (IQR 56–65)	M: 74 (35%), F: 139 (65%)	127–128 (IQR 117–139)	77–80 (IQR 73–87)	Normo: 25%; Controlled: 20%; Uncontrolled: 31%; Untreated: 25%	Median 31.2 (IQR 27–36.8)	DM: 21%
Espinel et al. (2023) [24]	30	N/A	59.6 ±8.8	M: 22 (66%), F: 8 (34%)	138.3 ±20.1	85.7 ±12.1	Resistant	33.1 ±4.4	DM: 36%; Dyslip: 76%; OSA: 60%; MetS: 63%; LVH: 90%; Plaque: 76%
Williams et al. (2015) [25]	Spiro: 285; Dox: 282; Biso: 285	Placebo: 274	61.4 ±9.6	M: 230 (69%), F: 105 (31%)	Home 147.6 ±13.2; Clinic 157.0 ±14.3	Home 84.2 ±10.9; Clinic 90.0 ±11.5	Resistant	N/A	DM: 46 (14%)
Lonn et al. (2016) [26]	6,356 (Cand+HCTZ)	6,349 (Placebo)	65.7 ±6.4	M: 6,831 (53.8%), F: 5,874 (46.2%)	138.1 ±14.7	81.9 ±9.3	Mild/intermediate risk	27.1 ±4.8	HTN: 38%; DM: 6%; Dysgly: 13%; Low HDL: 36%; Smoking: 28%; Renal: 2.8%
Fuchs et al. (2016) [27]	372 (51%)	358 (49%)	50 ±10 (Int), 50 ±11 (Ctrl)	Int: M/F 186/186; Ctrl: 179/179	127.9 ±7.3 vs 126.6 ±9.0	80.6 ±6.4 vs 80.4 ±6.2	Pre-HTN (SBP 120–139; DBP 80–89)	29 ±5	DM: 8%; Obesity: 32 vs 31%
Zhang et al. (2020) [28]	Enalapril+FA: 7,489 (50%)	Enalapril only: 7,489 (50%)	59 (45–75)	M: 6,110 (40.8%), F: 8,868 (59.2%)	SBP: 147.6–175.6 (by subgroup)	DBP: 90.2–96.0	Primary	24.6–25.1	Smoking: 16–25%; Alcohol: 15–25%; FamHx DM: 3–5%
Zhang et al. (2021) [29]	4,243 (49.9%)	4,268 (50.1%)	66.2 ±4.8	M: 3,960 (46.5%), F: 4,551 (53.5%)	146.1 ±16.8 vs 146.0 ±16.5	82.7 ±10.6 vs 82.3 ±10.5	Essential	25.5 ±3.2 vs 25.6 ±3.2	DM: 19.1%; CVD: 6.3%; HL: 36.8%; Renal: 2.4%
Mackenzie et al. (2022) [30]	Evening: 10,503	Morning: 10,601	65.1 ±9.3	M: 12,136 (57.5%), F: 8,968 (42.5%)	135.0 ±13.3 vs 134.8 ±13.3	79.1 ±9.2 vs 78.8 ±9.3	General HTN	28.4 ±4.8–4.9	CVD: 13%; DM: 13%; CKD: 3%; COPD: 3%; Asthma: 10%
Ishani et al. (2024) [31]	CTD: 6,756	HCTZ: 6,767	Median 72 (IQR 69–75)	M: 13,092 (96.8%), F: 431 (3.2%)	136 (IQR 129–146)	N/A	General HTN	30–31 (IQR 27–35)	DM: 42–54%; HF: 7–18%; CKD: 23–31%; Prior MI/stroke: 10.8%

The mean age of the participants ranged from 40.8 years in Akbarpour et al.’s study [11] to 72 years in Ishani et al.’s study [31]. Younger cohorts included Kimani et al.’s study [12], where 40.2% were under 50, while older populations were seen in Bertani et al.’s study [19] and Lonn et al.’s study [26] with means above 65. The gender distribution was generally balanced, while some studies such as Ishani et al.’s study [31] included a predominantly male (96.8%) population.

The baseline SBP was highest in the study by Zhang et al. [28], with subgroup means up to 175.6 mmHg, while the lowest was in the study by Fuchs et al. [27] at 126.6 mmHg. DBP values ranged from 73 mmHg in Gupta et al.’s study [23] to 96 mmHg in Zhang et al.’s study [28]. Hypertension type varied, with essential hypertension reported in the studies by Kimani et al. [12], Xiao et al. [13], and Unda Villafuerte et al. [17], stage 1-2 reported in the study by Villarino et al. [22], resistant hypertension reported in the studies by Blumenthal et al. [21] and Espinel et al. [24], and elderly hypertension reported in the study by Bertani et al. [19].

Moreover, BMI data showed variation, from 24.6 kg/m² in Zhang et al.’s study [28] to 36 kg/m² in Blumenthal et al.’s study [21]. Comorbidities were common: diabetes ranged from 1% in Hinderliter et al.’s study [20] to 54% in Ishani et al.’s study [31]; obesity was frequent, reported in up to 53% in Unda Villafuerte et al.’s study [17]; and cardiovascular disease prevalence reached 39.9% in Lu et al.’s study [14].

In terms of comorbidities, the diabetes prevalence was as low as <1% in Hinderliter et al.’s study [20] but reached 54% in Ishani et al.’s study [31]. Cardiovascular disease was frequent, with 19-39.9% reported in Lu et al.’s study [14] and 11% in Blumenthal et al.’s study [21]. Dyslipidemia ranged from 36.8% in Zhang et al.’s study [29] to 76% in Espinel et al.’s study [24]. Obesity was common, notably ~53% in Unda Villafuerte et al.’s study [17], while smoking was prevalent in 28% in Lonn et al.’s study [26]. More severe profiles were noted in Espinel et al.’s study [24], with 90% left ventricular hypertrophy and 76% carotid plaque.

Characteristics of Intervention

Table 3 shows the pharmacological and non-pharmacological characteristics of the participants. Notably, regarding the drug classes, there is a wide range of antihypertensives reported in the included studies. The studies by Akbarpour et al. [11] and Langford et al. [15] provided lifestyle data only, while Kimani et al. [12] documented ACE inhibitors, ARBs, diuretics, CCBs, BBs, and thiazides. The more detailed regimens were noted in the study by Blumenthal et al. [21], with thiazides (71%), loop diuretics (12%), mineralocorticoid receptor antagonists (21%), BB (72%), ARB (52%), CCB (77%), and ACEi (40%). Multiple-drug use was frequent, with Ishani et al. [31] reporting three agents, while Williams et al. [25] required ≥3 drugs in resistant cases. The dosage varied from fixed combinations, such as candesartan 16 mg with hydrochlorothiazide 12.5 mg in Lonn et al.’s study [26], to titrated regimens in Williams et al.’s study [25]. The duration of pharmacological intervention ranged from 12 weeks in Bertani et al.’s study [19] to over five years in Mackenzie et al.’s study [30].

**Table 3:** Characteristics of both pharmacological and non-pharmacological interventions ACEi, angiotensin-converting enzyme inhibitor; AldoAnt, aldosterone antagonist; Amil, amiloride; ARB, angiotensin receptor blocker; ASA, acetylsalicylic acid; BASNEF, Belief, Attitude, Subjective Norm, Enabling Factors; BB, beta-blocker; CA, combined aerobic; CCB, calcium channel blocker; C-LIFE, Comprehensive Lifestyle Intervention; COMBI, combined intervention; CTD, chlorthalidone; DASH, Dietary Approaches to Stop Hypertension; DDD, defined daily dose; Diur, diuretic; FA, folic acid; HCTZ, hydrochlorothiazide; HR, heart rate; IA, isolated aerobic; IPAQ, International Physical Activity Questionnaire; IQR, interquartile range; MRA, mineralocorticoid receptor antagonist; N/A, not applicable; Na, sodium; PA, physical activity; R, resistance training; RAAS, renin–angiotensin–aldosterone system; SEPA, Standard Education Plus Activity; SGLT2i, sodium–glucose cotransporter-2 inhibitor; Thiazide, thiazide diuretic; WM, weight management

	Pharmacological Interventions				Non-Pharmacological Interventions						
	Drug Class	Number of Drugs Used (N, %)	Dosage/Regimen	Duration	Diet	Exercise	Weight Loss	Smoking Cessation	Alcohol Reduction	Stress/Meditation	Non-pharm Duration
Akbarpour et al. (2018) [11]	N/A	N/A	N/A	N/A	Lifestyle score (fruits, veg, dairy, fast food, drinks, salt, oil)	Poor <30 min/wk; Mod 30–180; Good >180	N/A	Non-smokers 87.4% (aware 89.9%, not aware 85.8%)	N/A	N/A	N/A
Kimani et al. (2019) [12]	ACEi, ARB, diuretics, CCB, BB, thiazides	N/A	N/A	N/A	Veg daily 75.5%, fruit 44.1%, animal fat 31%, no fast food 55.5%	N/A	N/A	8.3% smoked; 94.4% never quit attempt	13.1% drinkers	N/A	N/A
Xiao et al. (2020) [13]	Antihypertensives	N/A	N/A	1 yr	Low salt/oil, veg, fruit, milk, poultry, fish 1–2/wk	Exercise ≥3 h/wk; daily strength	N/A	Smoking ↓ in intervention	Alcohol ↓ in intervention	N/A	1 yr
Lu et al. (2022) [14]	Antihypertensives	N/A	N/A	N/A	Lifestyle score (diet comp.)	Lifestyle score (activity)	N/A	Lifestyle score (non-/ex-/current smoker)	N/A	Stress in sensitivity analysis	N/A
Langford et al. (2024) [15]	N/A	N/A	N/A	N/A	50% knew DASH; 69% tried it	N/A	N/A	N/A	N/A	N/A	N/A
Marin-Couture et al. (2024) [16]	Antihypertensive	N/A	N/A	6 mo	DASH (NUT n=15, COMBI n=15 w/PA)	Aerobic ≥4 d/wk 30–60 min; strength ≥2/wk; 10k steps	N/A	Excluded smokers	N/A	N/A	6 mo
Unda Villafuerte et al. (2024) [17]	ACEi 40%, Diur 22.6%, ARB 15.7%, CCB 13.6%, BB 7.2%	Mean 1.7–1.8	N/A	6 mo	DASH 100%	Walking, 10–50 min, 3–7 d/wk (by group)	−0.9 kg (NS)	N/A	N/A	N/A	6 mo
Yang et al. (2017) [18]	ARB (Atacand)	Only 696 (61.1%), +1 351 (30.8%), +≥2 81 (7.1%)	N/A	12 wks	Salt normal 70%, high 26.6%	IPAQ: inactive 28.8%, min 17.5%, health 19.7%	N/A	Never 65.8%, ever 31%	32.3% drinkers	N/A	12 wks
Bertani et al. (2018) [19]	Antihypertensive	N/A	N/A	12 wks (36 sessions)	N/A	CA: 30 min 70%HR; IA: 30 min 60–80%; R: 9 ex, 2 sets, 6–10 reps	N/A	N/A	Excluded >7 drinks/wk	N/A	12 wks
Hinderliter et al. (2021) [20]	None	N/A	N/A	N/A	DASH	Aerobic 3/wk; 10 min warm, 30 min 70–85%HR, 5 min cool	−8.7 kg (DASH+WM)	N/A	N/A	N/A	16 wks
Blumenthal et al. (2021) [21]	Thiazide 71%, Loop 12%, MRA 21%, BB 72%, ARB 52%, CCB 77%, ACEi 40%	3.5 ±0.7	DDD 5.0 ±2.0	4 mo	DASH, Na ≤2,300 mg	Aerobic 3/wk, 30–45 min 70–85% HR	−6.9 kg (C-LIFE); −3.9 kg (SEPA)	4% smokers	Excl >14 drinks/wk	Counseling	4 mo
Villarino et al. (2021) [22]	N/A	N/A	N/A	N/A	Low Na	Lifestyle mod	N/A	N/A	N/A	BASNEF sessions	6 mo
Gupta et al. (2023) [23]	ACE/ARB 27%, BB 8%, CCB 18%, Diur 14%	0 drugs 51%, 1 drug 30%, 2 drugs 15%, ≥3 drugs 3%	N/A	1 wk diet arm	Low Na ~500 mg/d; High Na +2,200 mg	N/A	N/A	N/A	N/A	N/A	1 wk
Espinel et al. (2023) [24]	Diur, α/β-blockers, RAAS inhibitors	≥3	N/A	6 mo	Hypocaloric, low salt, DASH-like	Walk 45–60 min, resistance, step count	N/A	Non-smokers	N/A	Psych support	6 mo
Williams et al. (2015) [25]	ACE/ARB+CCB+Thiazide; Spiro, BB, α1-blocker	≥3	Spiro 25–50, Dox 4–8, Biso 5–10 (titrated)	12 wks/cycle	N/A	N/A	N/A	7.8% smokers	N/A	N/A	N/A
Lonn et al. (2016) [26]	ASA 11%, BB 8%, CCB 15%, α-blocker 1%, Diur 0.5%, AldoAnt 0.1%	2 drugs (fixed combo)	Cand 16 + HCTZ 12.5 daily	5.6 yrs	Lifestyle advice	N/A	N/A	N/A	N/A	N/A	N/A
Fuchs et al. (2016) [27]	Thiazide-like (CTD) + Amil	2 (combo)	CTD 12.5 + Amil 2.5 daily	18 mo	DASH (3 mo counsel)	Activity counseling	N/A	Smokers 8% vs 10%	Alcohol 61% vs 58%	N/A	3 mo
Zhang et al. (2020) [28]	Enalapril ± FA	1	Enalapril 10 ± FA 0.8	4.5 yrs	N/A	N/A	N/A	N/A	N/A	N/A	N/A
Zhang et al. (2021) [29]	Olmesartan, Amlodipine, HCTZ	1.9 (intensive), 1.5 (standard)	N/A	3.34 yrs (med)	N/A	N/A	N/A	N/A	N/A	N/A	N/A
Mackenzie et al. (2022) [30]	ACEi, ARB, BB, CCB, Diur, α-blocker	Mean 1.5 ±0.7	N/A	5.2 yrs	N/A	N/A	N/A	N/A	N/A	N/A	N/A
Ishani et al. (2024) [31]	HCTZ 25/50, ACEi/ARB 64–70%, Loop 2–3%, SGLT2i 2–5%, MRA 12–16%	Median 3 (IQR 2–4)	HCTZ 25/50; CTD 12.5/25 daily	2.4 yrs	N/A	N/A	N/A	22–32% smokers	N/A	N/A	N/A

Dietary modifications were common, with the DASH emphasized in the studies by Marin-Couture et al. [16], Hinderliter et al. [20], and Blumenthal et al. [21], while sodium restriction was central in the studies by Gupta et al. [23] and Villarino et al. [22]. Exercise prescriptions ranged from structured aerobic and resistance training [16,24] to walking programs [17]. Weight loss effects varied, with Hinderliter et al. [20] reporting a reduction of 8.7 kg in the DASH+WM arm and Blumenthal et al. [21] observing a reduction of 6.9 kg. Smoking cessation support was inconsistently reported, with Akbarpour et al. [11] noting high baseline non-smoking (87.4%) and Blumenthal et al. [21] reporting only 4% smokers. The alcohol reduction was included in the studies by Kimani et al. [12] and Fuchs et al. [27], with exclusion of heavy drinkers in Bertani et al.’s study [19]. Stress reduction and meditation were addressed in the studies by Villarino et al. [22] and Blumenthal et al. [21]. Non-pharmacological intervention duration paralleled trial length, ranging from one week in Gupta et al.’s study [23] to 5.6 years in Lonn et al.’s study [26].

Table 4 shows BP and clinical outcomes across included studies. The reductions in SBP were consistently observed, though the magnitude varied. The lifestyle awareness in Iran study showed the modest declines (-2.7 mmHg) [11], while the pharmacological and lifestyle interventions achieved larger reductions in Kenya (138.4 vs 140.1 mmHg) [12]. The most pronounced improvements were seen in China (-14.0 ± 13.5 mmHg) [13], Spain (-11.9 ± 17.0 mmHg) [17], and the USA (-16.1 mmHg with DASH+WM) [20]. Smaller reductions were noted in Brazil (-2 to -3 mmHg) [27]. Furthermore, DBP followed a similar trend, with decreases ranging from -1 mmHg [27] to nearly -10 mmHg [20].

**Table 4:** Blood pressure and clinical outcomes among the included studies AEs, adverse events; ABPM, ambulatory blood pressure monitoring; BMI, body mass index; CA, combined aerobic; C-LIFE, comprehensive lifestyle intervention; COMBI, combined intervention; CTD, chlorthalidone; CV, cardiovascular; DASH, Dietary Approaches to Stop Hypertension; EQ-VAS, EuroQol Visual Analogue Scale; HCTZ, hydrochlorothiazide; HDL/LDL, high-density/low-density lipoprotein; Hosp, hospitalization; HypoK, hypokalemia; IA, isolated aerobic; LS, lifestyle; MACE, major adverse cardiovascular events; Med, medication; NUT, nutrition; QoL, quality of life; R, resistance training; SEPA, Standard Education Plus Activity; target BP, target blood pressure; TC, total cholesterol; TG, triglycerides; UC, usual care; WM, weight management; ΔDBP, change in diastolic blood pressure; ΔHbA1c, change in glycated hemoglobin; ΔLipids, change in lipid profile; ΔSBP, change in systolic blood pressure

	ΔSBP (mean, SD)	ΔDBP (mean, SD)	Target BP (n, %)	ABPM (mean, SD)	Mortality (n, %)	CV Events (n, %)	Hosp. (n, %)	AEs (n, %)	ΔBMI/Weight (mean, SD)	ΔLipids (mean, SD)	ΔHbA1c (mean, SD)	QoL (mean, SD)	Adherence (n, %)
Akbarpour et al. (2018) [11]	Aware vs not aware: –2.7 mmHg	–7.5 mmHg	~30% controlled (non-med lifestyle)	N/A	N/A	N/A	N/A	N/A	N/A	N/A	N/A	N/A	Good lifestyle adherence: 28.5% overall
Kimani et al. (2019) [12]	Med+LS: 138.4 vs Med only: 140.1	85.1 vs 88.9 mmHg	34.8% (78/224) controlled	N/A	N/A	N/A	N/A	N/A	BMI 27.9 (controlled) vs 29.5 (uncontrolled)	Lower TC with fruit intake	N/A	N/A	85.2% took meds; 77.7% never forgot
Xiao et al. (2020) [13]	Int: –14.0 ± 13.5; Ctrl: –8.0 ± 18.4	Int: –9.7 ± 8.5; Ctrl: –5.5 ± 10.4	Int: 70.6%; Ctrl: 36.7%	N/A	N/A	N/A	N/A	N/A	N/A	TC, TG, HDL improved	N/A	N/A	Int: 87.6% at 1 yr vs Ctrl: 57.2%
Lu et al. (2022) [14]	N/A	N/A	N/A	N/A	2015 deaths	761 CVD deaths	N/A	N/A	N/A	N/A	↓ HbA1c with lifestyle	N/A	74% consistent med use
Langford et al. (2024) [15]	N/A	N/A	N/A	N/A	N/A	N/A	N/A	N/A	N/A	N/A	N/A	N/A	N/A
Marin-Couture et al. (2024) [16]	MED: –4.1; PA: –2.2; NUT: –6.0; COMBI: +3.6	MED: –1.5; PA: –2.3; NUT: –2.1; COMBI: +1.7	N/A	24-h ABPM baseline	N/A	N/A	N/A	N/A	MED: +0.5 kg; PA: –0.7 kg; NUT: –1.5 kg; COMBI: –0.8 kg	Lipid changes vary by group	HbA1c 5.5–5.9% baseline	N/A	89% completion
Unda Villafuerte et al. (2024) [17]	Int: –11.9 ± 17.0; Ctrl: –0.2 ± 15.4	Int: –5.9 ± 8.2; Ctrl: +0.7 ± 7.8	Int: 54.4%; Ctrl: 32.9%	N/A	N/A	N/A	N/A	4 AEs (dizziness, pruritus, edema)	Int: –0.6 (–2.5 to 1.4)	N/A	N/A	EQ-VAS ↑ (p=0.041)	Med adherence similar (77–79%)
Yang et al. (2017) [18]	N/A	N/A	84.4% overall (M 75.7%, F 90.0%)	N/A	N/A	N/A	N/A	N/A	N/A	N/A	N/A	N/A	≥80%: 93.0%
Bertani et al. (2018) [19]	CA: –4.0; IA: –2.7; R: +1.3; C: –2.9	CA: –3.6; IA: –2.0; R: +0.7; C: –0.9	N/A	24-h ABPM	N/A	N/A	N/A	N/A	N/A	N/A	N/A	N/A	87.1% completion
Hinderliter et al. (2021) [20]	DASH+WM: –16.1; DASH: –11.2; UC: –3.4	DASH+WM: –9.9; DASH: –7.5; UC: –3.8	Meds ↓ (e.g., DASH+WM 51→18%)	N/A	N/A	N/A	N/A	N/A	–8.7 kg (DASH+WM)	LDL ↓ more with DASH+WM	N/A	N/A	N/A
Blumenthal et al. (2021) [21]	C-LIFE: –12.5; SEPA: –7.1	C-LIFE: –5.9; SEPA: –3.7	C-LIFE: 59%; SEPA: 38%	24-h SBP ↓ in C-LIFE	N/A	N/A	N/A	N/A	C-LIFE: –2.3; SEPA: –1.3	LDL ↓ (–4.8 to –7.8); TG ↓	HbA1c ~ –0.3	N/A	Adherence 89–97%
Villarino et al. (2021) [22]	146.5→134.9 (–11.6)	84.6→78.8 (–5.8)	N/A	N/A	N/A	N/A	N/A	N/A	N/A	N/A	N/A	N/A	N/A
Gupta et al. (2023) [23]	–8 mmHg	–3 mmHg	73.4% had BP decline (LS diet)	ABPM: SBP –7; DBP –2	N/A	N/A	N/A	9–10% mild AEs	N/A	N/A	N/A	N/A	Urine sodium used
Espinel et al. (2023) [24]	–14.0 (138.3→124.3)	–8.5 (85.7→77.2)	80% responders	24-h SBP/DBP ↓ significantly	N/A	N/A	N/A	None	↓ BMI	Dyslipidemia 76%	N/A	MoCA unchanged	Compliance moderate
Williams et al. (2015) [25]	–8.7 mmHg	–3.9 mmHg	58% with spirono; overall 68.9%	N/A	N/A	N/A	N/A	19–23% AEs, 2% serious	N/A	N/A	N/A	N/A	High adherence
Lonn et al. (2016) [26]	–6.0 ± 13.0 vs placebo	–3.0 ± 8.0 vs placebo	Subgroup benefit if SBP >143.5	N/A	5.4% vs 5.5%	CV death, MI, stroke low	5.0% vs 5.2%	Symptomatic hypo ~3%	N/A	N/A	N/A	N/A	Adherence ~77%
Fuchs et al. (2016) [27]	–2 to –3 mmHg vs placebo	–1 mmHg vs placebo	N/A	N/A	N/A	N/A	N/A	AEs ~38%	N/A	LDL ↑; HDL ↑ slightly	N/A	N/A	Pill counts & self-report
Zhang et al. (2020) [28]	Tight: 126; Inter: 135; Poor: ≥148	Tight: 80–82; Poor: ≥96	N/A	N/A	N/A	N/A	N/A	N/A	N/A	N/A	N/A	N/A	N/A
Zhang et al. (2021) [29]	–19.4 (Intensive) vs –10.1 (Standard)	76.4 (Int) vs 79.2 (Std)	67–77% reached goal	N/A	1.6% vs 1.5%	3.5% vs 4.6%	N/A	Hypotension 3.4%; dizziness 1.1%	N/A	TC, LDL reported	N/A	N/A	95.8% tele-monitoring
Mackenzie et al. (2022) [30]	Morning vs evening: SBP diff ±1–2 mmHg	DBP diff ±1 mmHg	N/A	N/A	4.2% vs 4.1%	CV death/MI/stroke ~3–4%	Hosp 0.7–0.9%	AEs ~37–43%	N/A	N/A	N/A	N/A	~70% adhered
Ishani et al. (2024) [31]	No difference	N/A	N/A	N/A	CTD 8.9% vs HCTZ 12.2%	MACE diff by history of MI/stroke	Hosp ~26–37%	HypoK ~3–6%	N/A	N/A	N/A	N/A	N/A

The achievement of the target BP varied widely, with 34.8% in Kenya [12], 70.6% with intervention in China [13], 58-68.9% with spironolactone in the UK [25], and 67-77% under intensive regimens in China [29]. Ambulatory BP monitoring had confirmed the significant improvements in Spain and Brazil [19,24].

Clinical outcomes included mortality, reported in China (2015 deaths) [14] and the USA (CTD 8.9% vs HCTZ 12.2%) [31]. Cardiovascular events were modest, ranging from 3-4% in UK trials [30] to 3.5-4.6% in China [29]. Hospitalization rates were low (<1%) in Mackenzie et al.’s study [30] but higher (26-37%) in VA cohorts [31]. Adverse events ranged from 9-10% [23] to 43% [30], with hypotension and dizziness most frequently reported. Non-BP outcomes showed significant weight loss (-8.7 kg) [20], lipid improvements [13,21], HbA1c reductions [14,21], and better quality of life in Spain [17]. Adherence remained generally high, reaching 95.8% with tele-monitoring [29].

Meta-Analysis

**Figure 6:**
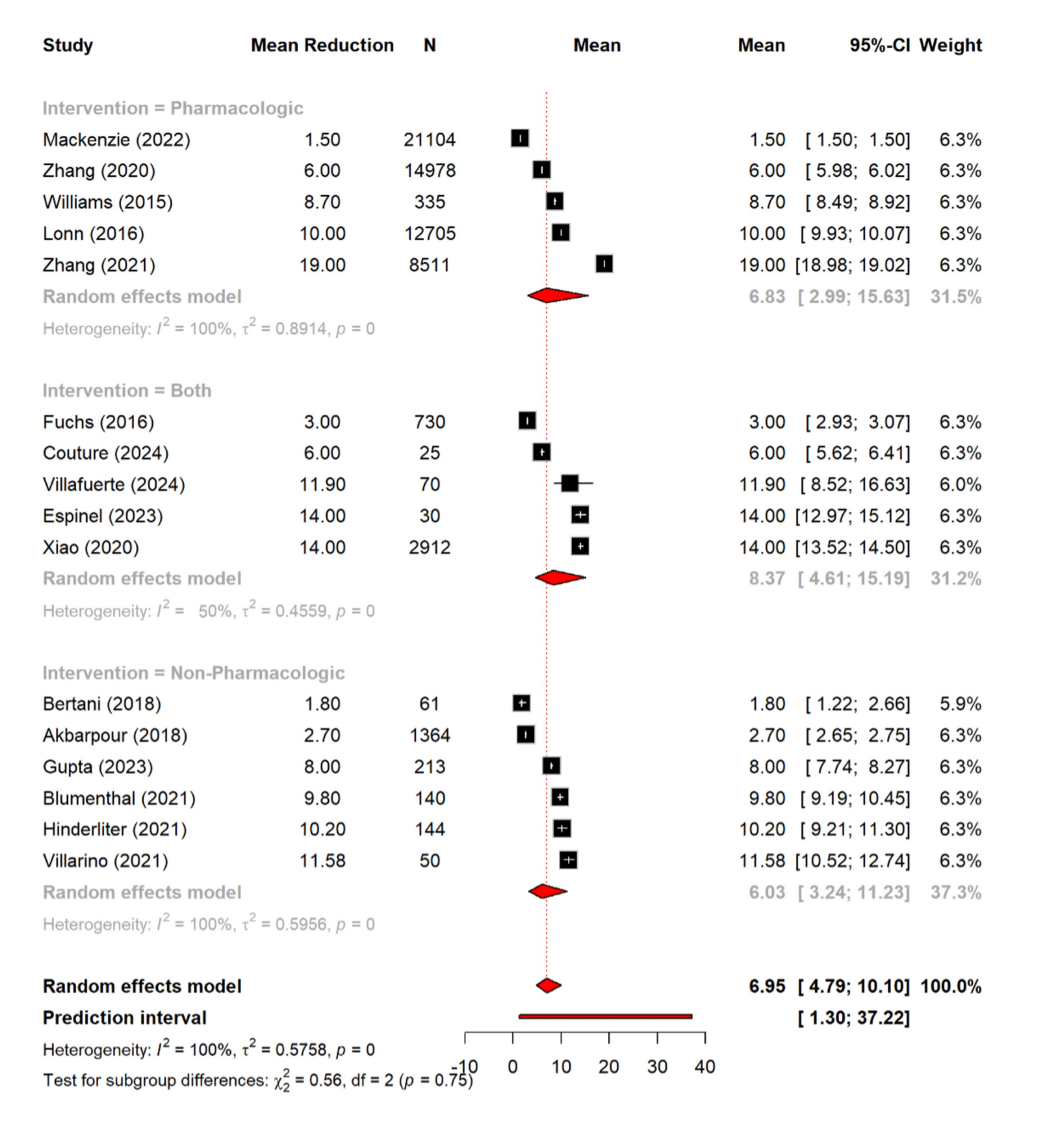
Mean reduction in systolic blood pressure based on different management types [11,13,16,17,19-25,27-30]

The meta-analysis of this study assessed the difference of different parameters based on management type. Notably, there is a pooled mean reduction of 6.95 mmHg (95% CI: 4.79-10.09) in SBP across 16 studies (n = 63,372) based on the random effect model. The subgroup analysis revealed that there is a comparable reduction of SBP among different types. The pharmacological interventions achieved a mean reduction of 6.83 mmHg (95% CI: 2.99-15.63), while the non-pharmacological interventions achieved a mean reduction of 6.03 mmHg (95% CI: 3.24-11.23). Moreover, the combined interventions achieved the greatest mean reduction of 8.37 mmHg (95% CI: 4.61-15.19). However, these differences between the different types of interventions were not statistically significant (Q = 0.56, p = 0.75) (Figure 6).

**Figure 7:**
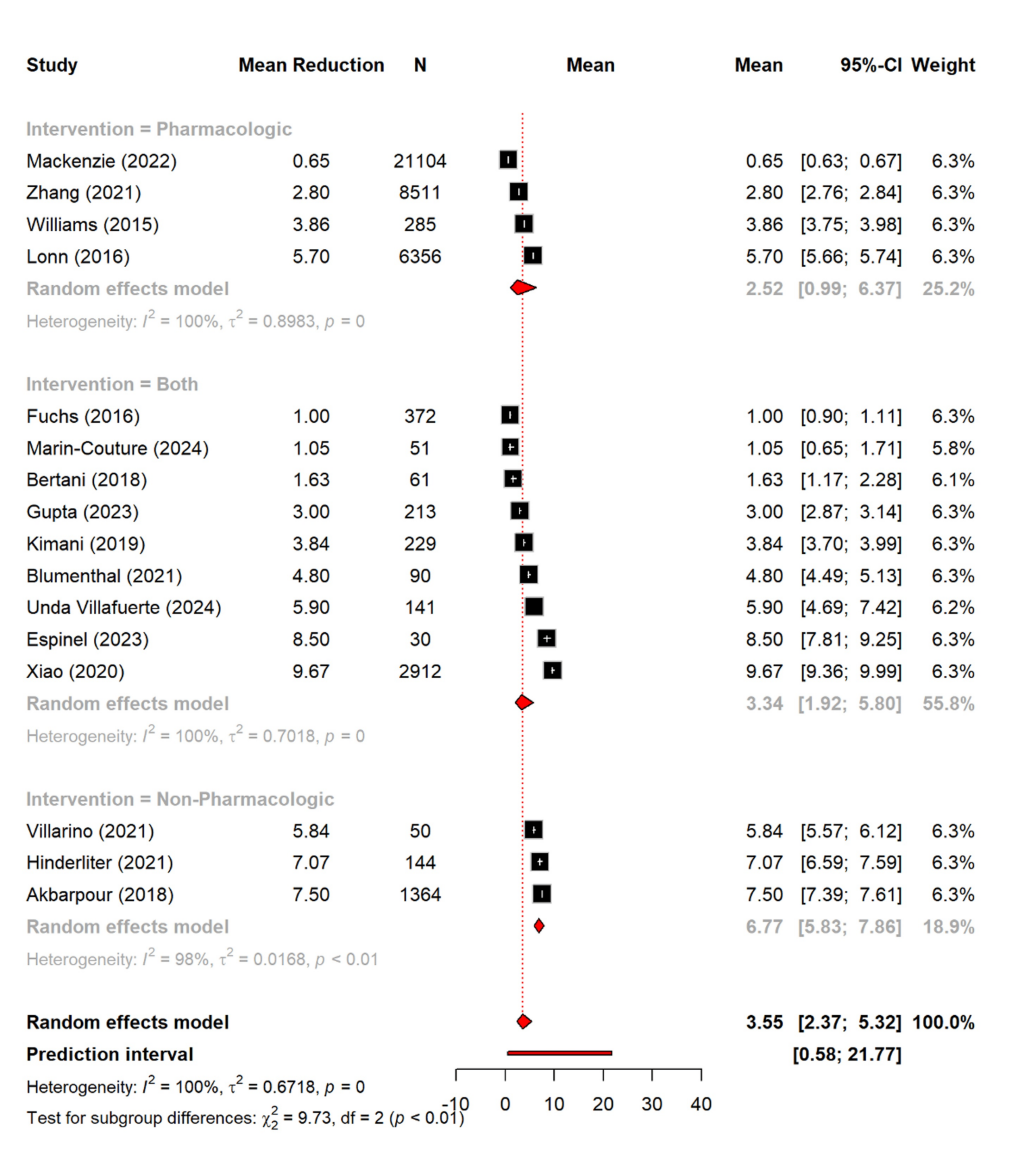
Mean reduction in diastolic blood pressure based on different management types [11-13,16,17,19-25,27-30]

In terms of DBP, the pharmacological interventions achieved a mean reduction of 2.52 mmHg (95% CI: 0.99-6.37), the combined approaches showed a reduction of 3.34 mmHg (95% CI: 1.92-5.80), and the non-pharmacological interventions showed the largest reduction with 6.77 mmHg (95% CI: 5.83-7.86). The subgroup differences were statistically significant (Q = 9.73, p = 0.0077) (Figure 7).

**Figure 8:**
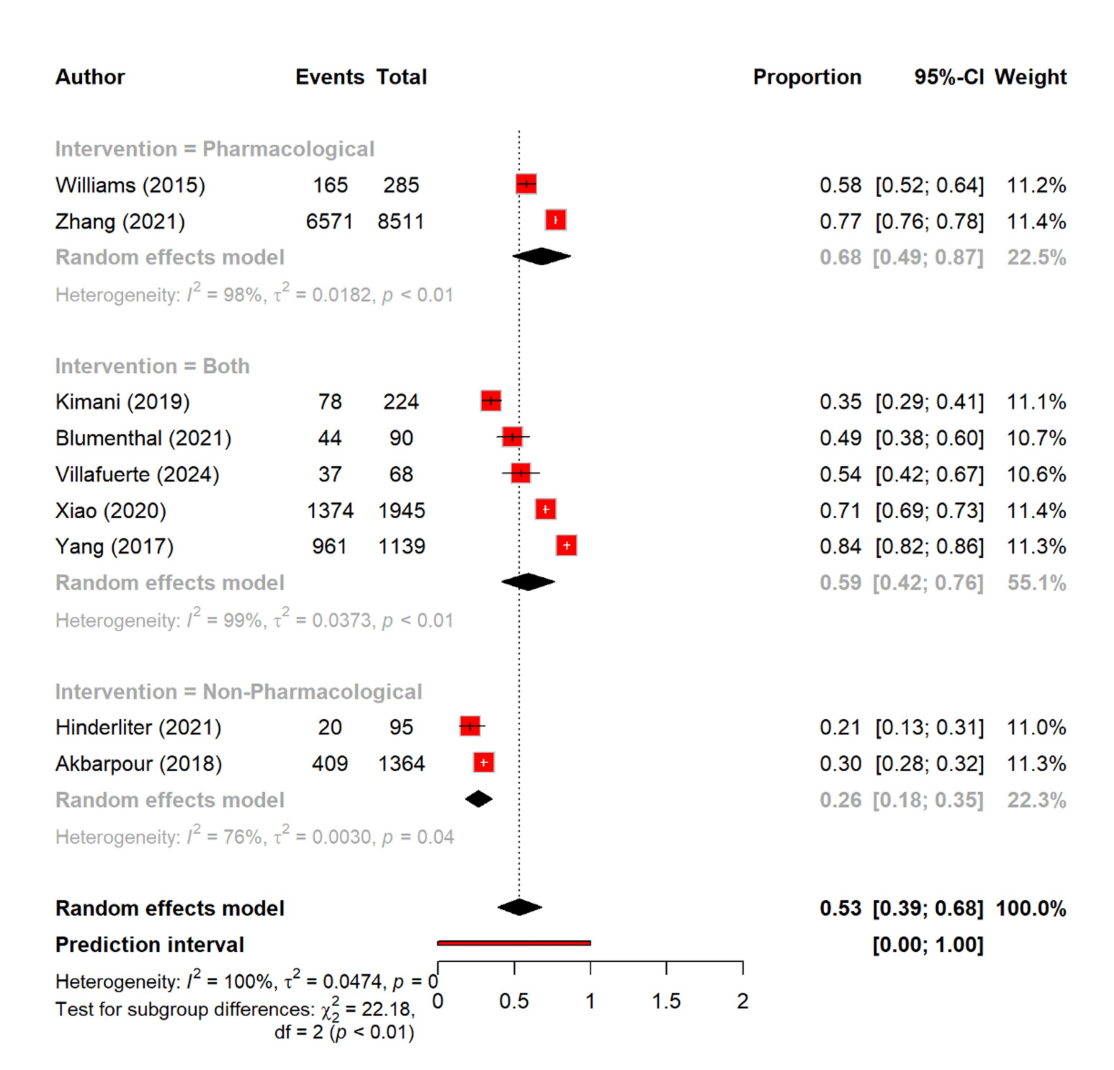
Proportion of targeted blood pressure based on different management types [11-13,16,17,19-25,27-30]

If we looked for the proportion of patients who achieved the target BP, the pooled rate was 53.4% (95% CI: 39.0-67.8). The pharmacological management yielded the highest proportion of 67.8%, which achieved their target range of BP (95% CI: 48.9-86.7). Furthermore, the combined interventions showed that 58.9% (95% CI: 41.7-76.2) of participants had achieved their target range of BP. For the non-pharmacological strategies, there were only 26.4% (95% CI: 17.8-35.0), which achieved their targeted range of BP. The subgroup differences were significant (Q = 22.18, p < 0.0001) (Figure 8).

**Figure 9:**
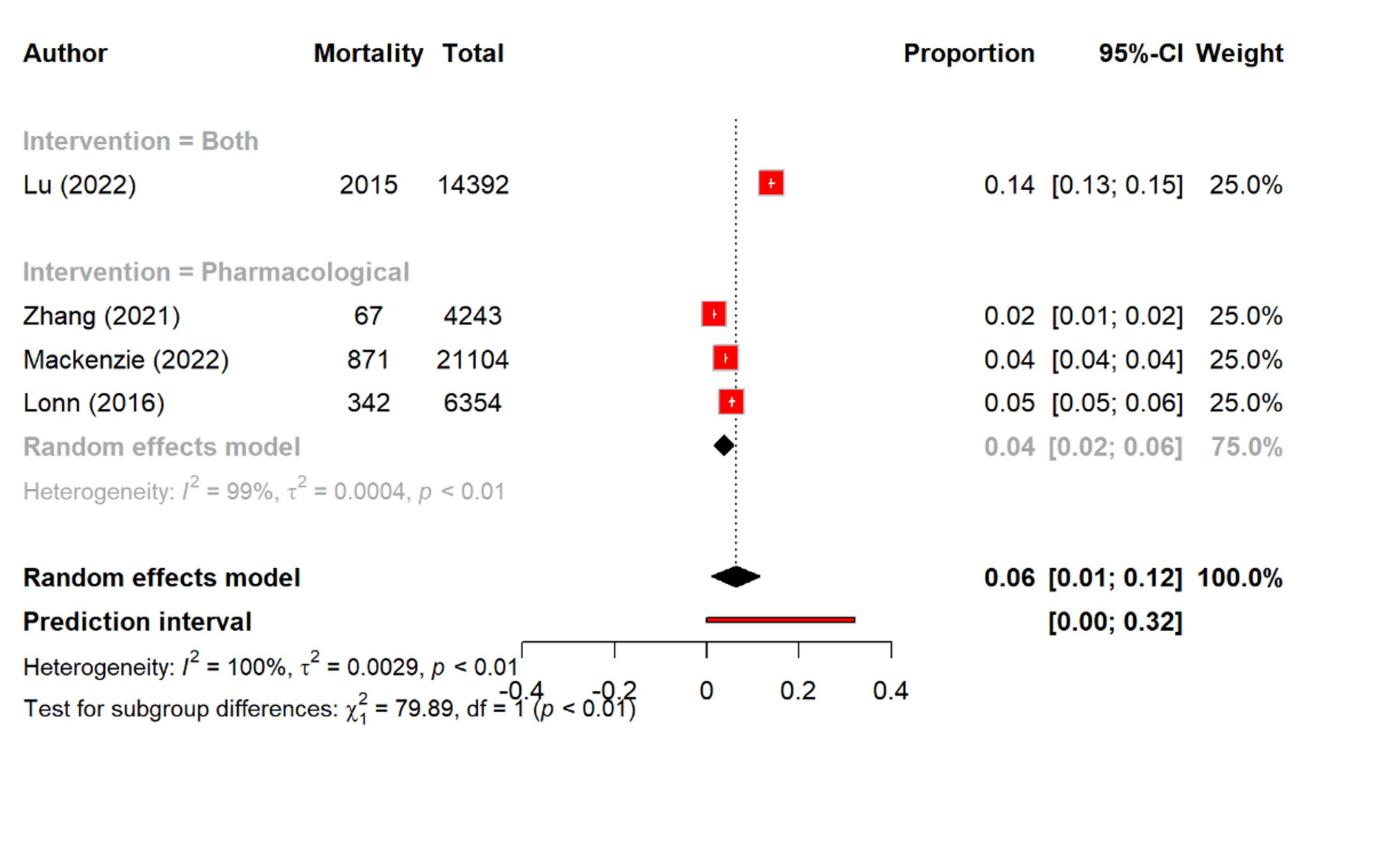
Overall mortality rate among different management types [14,26,29,30]

In terms of mortality, the pooled proportion was 6.3% (95% CI: 1.0-11.6). The pharmacological interventions showed that there was a lower mortality rate of 3.7% (95% CI: 1.5-5.9) in this group, while there was a higher rate of 14% in the combined intervention, but it was reported in only one study. The differences were significant between the groups (Q = 79.89, p < 0.0001) (Figure 9).

**Figure 10:**
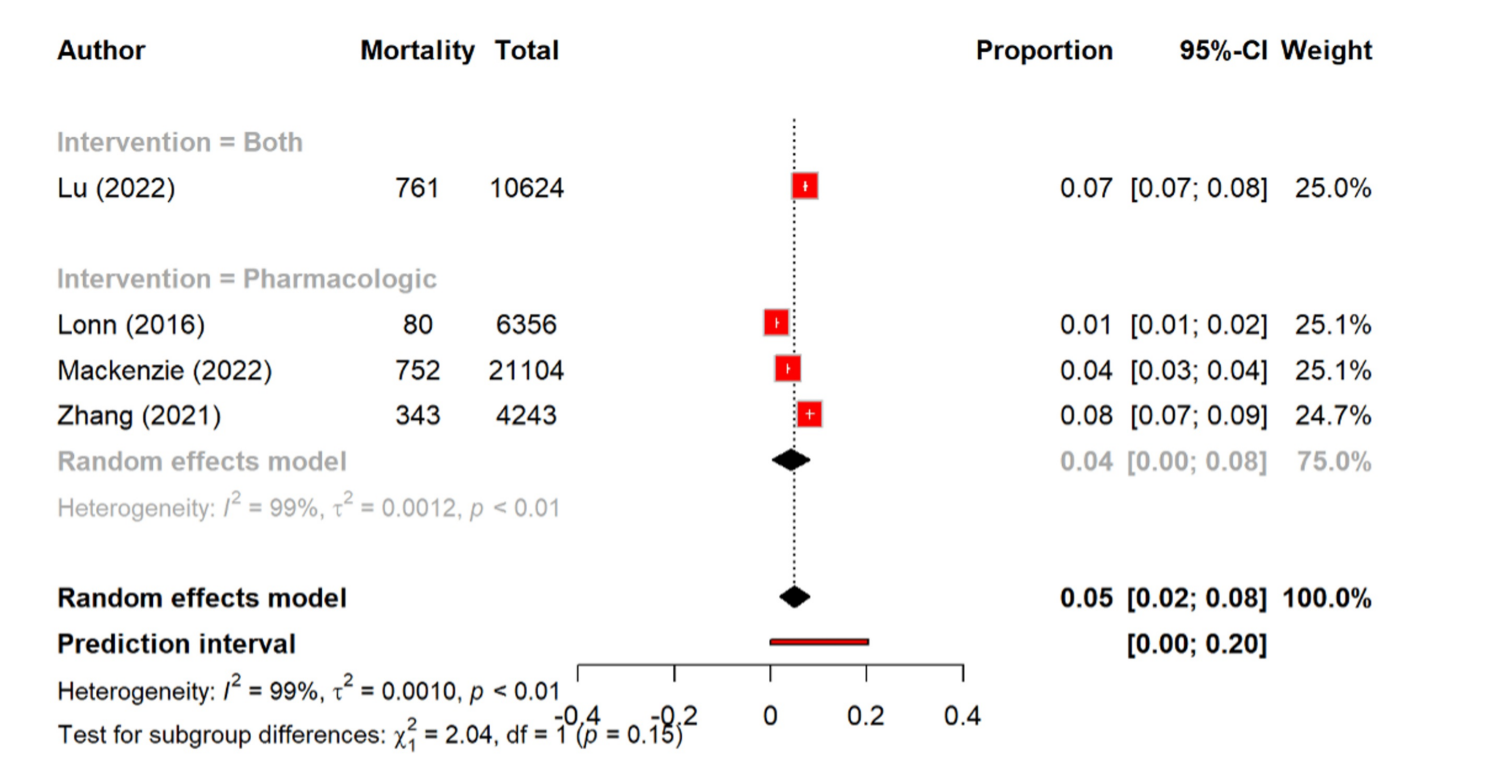
Overall cardiovascular mortality rate among different management types [14,26,29,30]

In terms of cardiovascular deaths, there was a mortality rate of 5.0% (95% CI: 1.9-8.1). The pharmacological management showed a pooled rate of 4.3% (95% CI: 0.4-8.2), as compared to 7.2%, in the single combined intervention study, and the subgroup differences were not significant (Q = 2.04, p = 0.15) (Figure 10).

**Figure 11:**
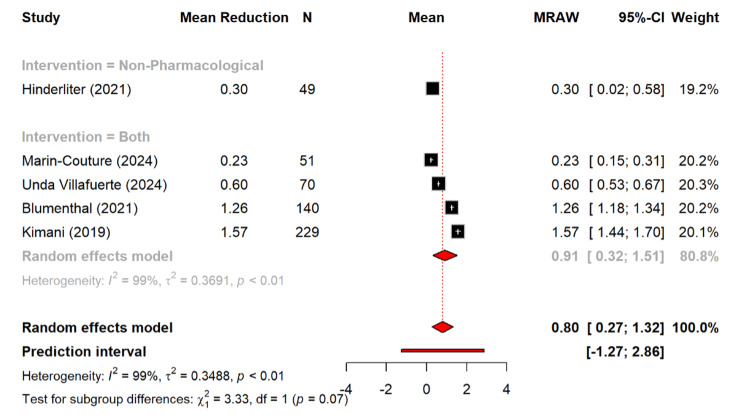
Reduction of mean BMI among different management types [12,16,17,20,21]

This study also assessed some secondary outcomes such as BMI and cholesterol level. For BMI, there was a pooled mean reduction of 0.80 kg/m² (95% CI: 0.27-1.32). The combined interventions produced a larger reduction of 0.91 kg/m² (95% CI: 0.32-1.51) as compared to a single non-pharmacological study reporting 0.30 kg/m². The subgroup differences approached significance (Q = 3.33, p = 0.07) (Figure 11).

**Figure 12:**
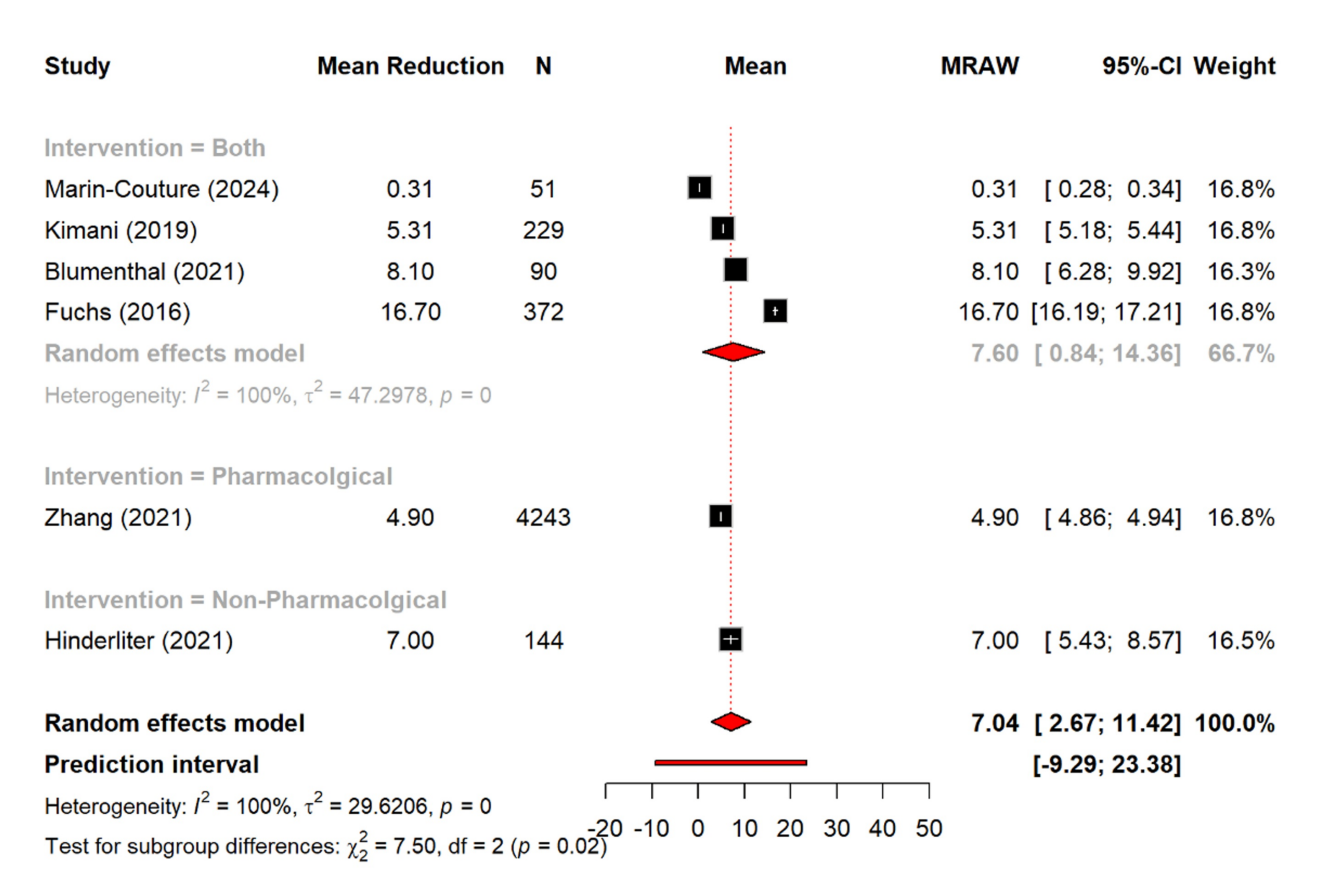
Reduction of mean total cholesterol among different management types [12,16,20,21,27,29]

Figure 12 shows the reduction of total cholesterol. Notably, there is a pooled mean reduction of 7.04 mg/dL (95% CI: 2.67-11.42). The combined interventions showed the largest reduction of 7.60 mg/dL (95% CI: 0.84-14.36), as compared to the non-pharmacological (7.00 mg/dL) and the pharmacological interventions (4.90 mg/dL from a single study). The subgroup differences were statistically significant (Q = 7.50, p = 0.0235).

**Figure 13:**
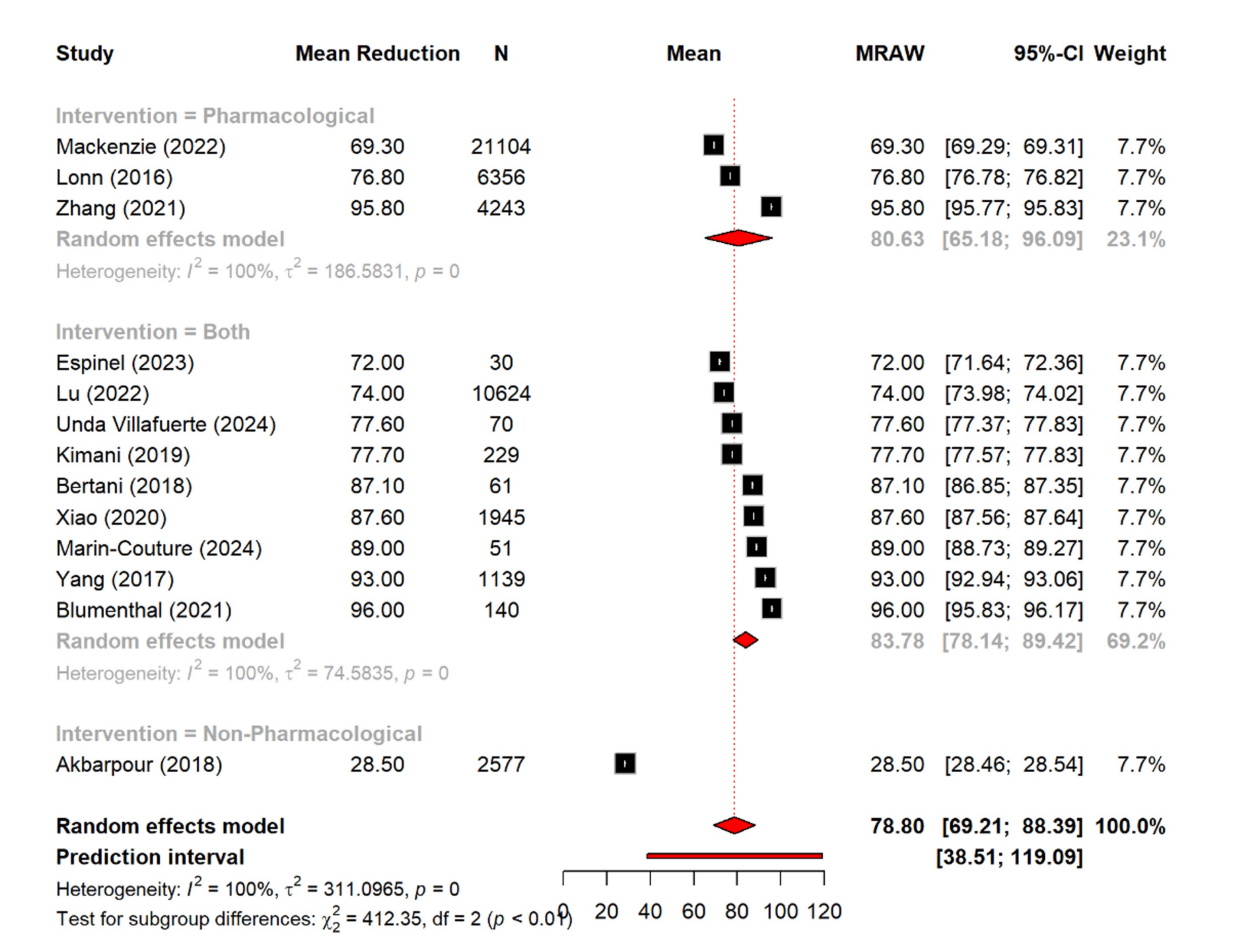
Reported compliance rate of different management types [11-14,16-19,21,24,26,29,30]

Finally, the overall compliance rate reported was 78.8% (95% CI: 69.2-88.4; I² = 100%). The studies with combined interventions demonstrated the highest compliance rate of 83.8% (95% CI: 78.1-89.4), followed by the compliance proportion in the studies with pharmacological intervention with 80.6% (95% CI: 65.2-96.1), while the study with non-pharmacological interventions showed markedly lower compliance (28.5% from a single study). Subgroup differences were significant (Q = 412.35, p < 0.0001) (Figure 13).

**Figure 14:**
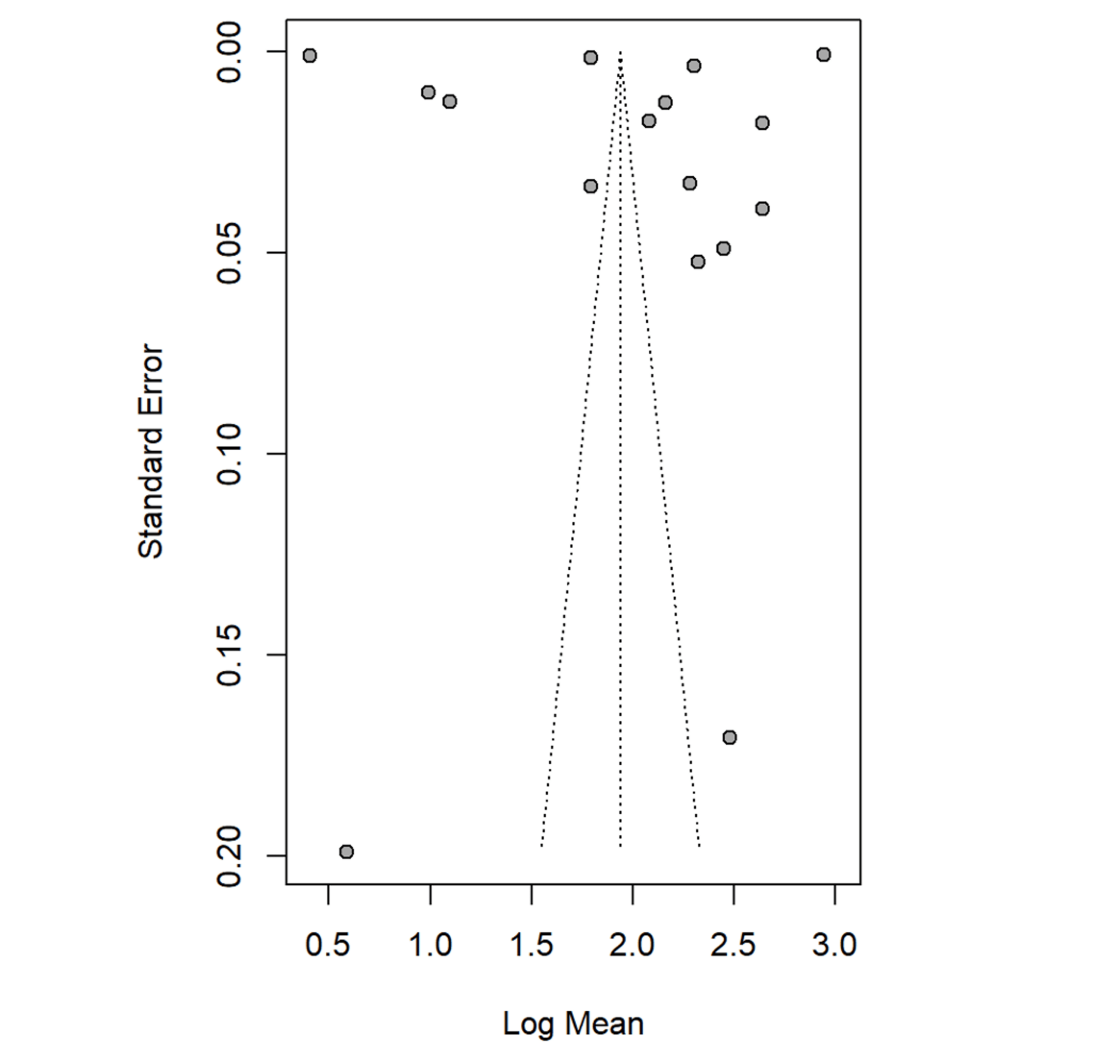
Assessment of publication bias

Figure 14 shows the funnel plot for the assessment of the publication bias. Ideally, the studies should be symmetrically distributed around the central line. In this plot, the asymmetry is evident, with several of the studies clustering more on the right side and fewer scattered on the left side. This suggests that there is a potential publication bias, where the smaller studies with negative or the non-significant results may be underrepresented. The spread of points at the top indicates larger studies with lower standard error, while the wider scatter at the bottom shows smaller studies with greater variability. Overall, the pattern raises concern about selective reporting or missing negative studies.

Discussion

Hypertension is one of the major health burdens worldwide and also the leading cause of preventable illness and death [32]. The pharmacological therapies, which included diuretics, ACE inhibitors, ARBs, CCBs, and BBs, are effective and first-line drugs for hypertension [33]. However, drug therapy is often limited by their side effects, cost, and adherence issues. The non-pharmacological strategies, which included dietary changes, exercise, weight management, salt reduction, and the stress control, provide comparable benefits with added advantages for the overall health [34]. This systematic review and meta-analysis aimed to compare the pharmacological, non-pharmacological mainly, and the combined interventions to guide evidence-based approaches for effective hypertension management and improved patient outcomes.

Notably, our study showed that the pharmacological interventions achieved reductions of 6.83 mmHg in SBP, the non-pharmacological strategies alone also reduced the SBP (6.03 mmHg), and the combined approaches yielded the largest reduction in SBP (8.37 mmHg). These findings highlight that the combined approach delivered the greatest improvement in lowering the SBP, which showed that there is a synergistic effect of integrating medications with lifestyle interventions. A study by Krishnamoorthy et al. showed that healthy diet and physical activity is the most important combination of lifestyle modifications for prehypertensive and hypertensive patients, along with the pharmacological management [35]. These findings reinforced the current guideline recommendations that lifestyle modification should also accompany drug therapy whenever possible. However, a study by Nasir et al. showed that pharmacological treatments resulted in faster and slightly greater reductions in SBP (mean SBP reduction: 9-14 mmHg), while lifestyle interventions produced sustained, clinically meaningful reductions (mean SBP reduction: 6-11 mmHg) [36]. The similarity of non-pharmacological and pharmacological effect sizes in our study underscores the clinical importance of non-drug strategies, particularly in low-resource or prevention settings.

For DBP, the subgroup differences were statistically significant, with the non-pharmacological approaches producing the largest mean reduction (6.77 mmHg). This result is aligned with the prior meta-analyses, including that by Chen et al., who found that the certain lifestyle combinations (diet plus Tai Chi or structured exercise) achieved DBP reductions exceeding -13.62 mmHg [37]. Our results add weight to the argument that well-implemented lifestyle interventions can equal or even surpass drug therapy in lowering DBP. However, the sustainability of these findings in real-world practice remains a challenge.

Furthermore, the achievement of target BP control provides a more clinically relevant measure than mean reductions. In our analysis, 53.4% of participants overall achieved the target thresholds. Pharmacological therapies were the most effective, with 67.8% reaching the target BP, followed by combined interventions at 58.9%, while non-pharmacological approaches were less successful (26.4%). In our study, these differences were statistically significant. These findings aligned with previous studies that indicate that drug therapy is the backbone of hypertension control and also highlights that lifestyle-only strategies may be insufficient for many patients, particularly those with the advanced disease or comorbidities. A study by Tocci et al. showed that 14.0% of participants achieved their target range of BP according to European guidelines with pharmacological therapy [38]. McGuire et al. showed that 12% in the DASH diet group achieved their target range of BP [39]. Similar conclusions were drawn in the ALLHAT and SPRINT trials, where pharmacological therapy achieved robust control rates, but adherence and adjunct lifestyle measures influenced long-term outcomes [40,41].

The mortality outcomes were less consistently reported but provide important insights into this context. Notably, overall mortality was 6.3%, with the pharmacological therapy showing the lowest rate (3.7%) of mortality as compared to the higher estimate in the sole combined-intervention study (14%). The cardiovascular mortality was 5.0% overall, with the pharmacological therapy again associated with the lower rates (4.3%) of mortality as compared to combined interventions (7.2%). Although the number of studies was small, these findings are consistent with the robust evidence base for the antihypertensive drugs in reducing cardiovascular disease and all-cause mortality. The lifestyle interventions, while effective in BP reduction, rarely demonstrate such mortality benefits unless combined with pharmacotherapy or sustained long-term. A study by Lu et al. showed that antihypertensive drugs plus favorable lifestyle lowered all-cause mortality (HR 0.32) and CVD (HR 0.33). Favorable lifestyle alone also reduced risks (HR 0.34, 0.40, 0.33), while the drugs with poor lifestyle showed no significant mortality reduction [14].

The secondary outcomes provide further insights. BMI reductions were modest (0.80 kg/m² overall), with combined interventions showing the largest effect (0.91 kg/m²). Similarly, a study by Torres et al. showed that patients in the dietary counseling group showed significantly greater reductions in body weight (-3.6±0.8) as compared to the control group [42]. The total cholesterol fell by 7.04 mg/dL, with significant subgroup differences (p = 0.0235). Furthermore, the combined strategies produced the largest improvements in total cholesterol, which reflected the additive value of diet, exercise, and pharmacological therapy, which is aligned with the previous study [43].

Strength and Limitations

The strengths of this review include the large aggregated sample, with the inclusion of both pharmacological and non-pharmacological interventions, and reporting of the multiple outcomes beyond the BP, including mortality and adherence. The limitations included the high heterogeneity (I² ~100%), with the varying study quality, short follow-up in many lifestyle trials, and the limited data on hard outcomes such as mortality in non-pharmacological arms. The publication bias and inconsistent adherence reporting are additional concerns.

Implications

Pharmacological therapy remains the most reliable method to achieve the target BP and reduce mortality, but lifestyle interventions add important complementary benefits, especially for DBP reduction, weight, and metabolic health. For patients with mild hypertension or high risk of adverse drug effects, structured non-pharmacological interventions may offer meaningful alternatives. At the population level, a combination of pharmacological therapy and accessible, sustainable lifestyle programs represents the optimal approach to improving control rates and reducing cardiovascular burden.

## Conclusions

This systematic review and meta-analysis demonstrate that both pharmacological and non-pharmacological interventions significantly reduce BP, with combined strategies producing the greatest effect. Pharmacological therapies achieved the highest rates of target BP control and lowest mortality, while non-pharmacological approaches, particularly lifestyle modifications, showed notable diastolic benefits and improvements in BMI and cholesterol. The compliance was reported highest in the studies with combined and pharmacological regimens, but limited in lifestyle-only strategies. Overall, integrating drug therapy with lifestyle measures appears most effective for sustainable hypertension control and reducing associated cardiovascular risks.
